# Caloric Restriction Enhances Chemotherapy Efficacy and Reshapes Stress Responses in Sarcoma

**DOI:** 10.3390/cancers18010110

**Published:** 2025-12-29

**Authors:** Jorddam Almondes Martins, Irislene Costa Pereira, Thiago Sousa Reinaldo, Dallyla Jennifer Morais de Sousa, Isabelle Vasconcelos Rodrigues, Beatriz de Mello Pereira Rego, Aureliano Machado de Oliveira, Taline Alves Nobre, Athanara Alves de Sousa, João Pedro Alves Damasceno do Lago, Rayran Walter Ramos de Sousa, Diego Pereira de Menezes, Alda Cássia Alves da Silva, Dalton Dittz, Adriana Maria Viana Nunes, Vladimir Costa Silva, Juliana Soares Severo, Moisés Tolentino Bento da Silva, Paulo Michel Pinheiro Ferreira, João Marcelo de Castro e Sousa, Francisco Leonardo Torres-Leal

**Affiliations:** 1 Metabolic Diseases, Exercise and Nutrition Group (DOMEN), Glauto Tuquarre Metabolic Diseases Laboratory (LabGT), Department of Biophysics and Physiology, Federal University of Piauí, Teresina 64049-550, PI, Brazil; jorddamalmondes@ufpi.edu.br (J.A.M.); irislenecosta@ufpi.edu.br (I.C.P.); thiago.reinaldo@ufpi.edu.br (T.S.R.); dallylamorais@ufpi.edu.br (D.J.M.d.S.); isabellevasconcelos@ufpi.edu.br (I.V.R.); beatrizz.mello@hotmail.com (B.d.M.P.R.); aureliano@frn.uespi.br (A.M.d.O.); aldacassiia.ads@ufpi.edu.br (A.C.A.d.S.); julianasevero@ufpi.edu.br (J.S.S.); 2 Toxicological Genetics Research Laboratory (LAPGENIC), Department of Biochemistry and Pharmacology, Federal University of Piauí, Teresina 64049-550, PI, Brazil; taline.nobre@ufpi.edu.br (T.A.N.); athanaraalves@ufpi.edu.br (A.A.d.S.); joao.lago@ufpi.edu.br (J.P.A.D.d.L.); j.marcelo@ufpi.edu.br (J.M.d.C.e.S.); 3 Laboratory of Experimental Cancerology (LABCANCER), Department of Biophysics and Physiology, Federal University of Piauí, Teresina 64049-550, PI, Brazil; rayran.ramos@ufpi.edu.br (R.W.R.d.S.); pmpf@ufpi.edu.br (P.M.P.F.); 4 Laboratory of Antineoplastic Pharmacology (LAFAN), Department of Biochemistry and Pharmacology, Federal University of Piauí, Teresina 64049-550, PI, Brazil; diegomenezes@ufpi.edu.br (D.P.d.M.); daltondittz@ufpi.edu.br (D.D.); 5 Pathology and Histology Laboratory (LaPaH), Department of Biophysics and Physiology, Federal University of Piauí, Teresina 64049-550, PI, Brazil; adriananunes@ufpi.edu.br; 6 Genomic Surveillance and Molecular Biology Laboratory, Oswaldo Cruz Foundation, Teresina 64049-550, PI, Brazil; vladimir.costa@fiocruz.br; 7 Laboratory of Physiology, Center for Drug Discovery and Innovative Medicines (MedInUP/RISE-Health): Health Research Network, Department of Immuno-Physiology and Pharmacology, School of Medicine and Biomedical Science, University of Porto, 4099-002 Porto, Portugal

**Keywords:** caloric restriction, doxorubicin, cancer, stress response, sarcoma-180

## Abstract

**Background:** Oncological treatment remains a major clinical challenge. Despite therapeutic advances and the diversity of available approaches, many tumors continue to exhibit limited responsiveness to chemotherapy. In this context, nutrition has emerged as a promising complementary strategy to support cancer therapy. In particular, interventions based on nutritional deprivation have gained prominence due to their ability to modulate tumor metabolism, inducing alterations that may increase the sensitivity of cancer cells to conventional treatments. Accordingly, the present study aimed to evaluate the safety and efficacy of caloric restriction combined with chemotherapy in a Sarcoma-180 model, investigating its effects on immunological and hematological parameters, antioxidant activity, oxidative stress, and tumor and liver morphology, as well as DNA damage. **Methods:** Mice bearing Sarcoma-180 were randomly assigned to four groups: Ad Libitum (AL), Ad Libitum + Doxorubicin (ALDOX), Caloric Restriction (CR), and Caloric Restriction + Doxorubicin (CRDOX). Assessment included tumor weight and volume, food and caloric intake, hematotoxicity, lipid metabolism, oxidative stress and antioxidant markers, genotoxicity, morphological alterations in the tumor and liver, and overall survival. **Results:** The data obtained demonstrate that caloric restriction combined with doxorubicin is both safe and feasible, as it preserves body weight and does not induce metabolic disturbances. Importantly, this combined strategy produced a marked reduction in tumor volume and mass while also mitigating the hematotoxicity typically associated with doxorubicin. In peripheral blood, the regimen decreased chemotherapy-induced DNA damage, supporting a systemic protective effect. Consistently, the combination reduced oxidative stress markers (NOx and MDA) and enhanced antioxidant activity within the tumor. Histological analyses further confirmed these outcomes, showing tumor cell death with features compatible with apoptosis and reduced local invasion. Together, these data indicate that caloric restriction enhances the antitumor efficacy of doxorubicin while simultaneously improving treatment tolerance. **Conclusions:** This study demonstrates that caloric restriction, combined with doxorubicin, is safe, well-tolerated, and enhances the antitumor response in the Sarcoma-180 model.

## Simple Summary

Cancer treatments often cause strong side effects and do not always stop tumor growth. Recent research suggests that reducing calorie intake for a short period may help the body respond better to chemotherapy while protecting healthy cells. In this study, it was investigated whether combining caloric restriction with the chemotherapy drug doxorubicin could enhance treatment outcomes in mice with sarcoma. It was found that this combined approach reduced tumor growth more effectively than chemotherapy alone and helped protect the blood, liver, and DNA from treatment-related damage. It also lowered oxidative stress and improved survival. These findings suggest that controlled caloric restriction may be a safe and promising approach to enhance the effects of chemotherapy and mitigate its adverse effects. This approach may guide future research on how nutrition can support cancer treatment.

## 1. Introduction

Cancer, characterized by the uncontrolled growth of abnormal cells, continues to pose a challenge to the scientific community despite extensive research [1,2,3,4,5]. The global prevalence of cancer and the obstacles associated with cellular resistance underscore the urgent need for innovative approaches to manage the disease [6,7,8,9,10].

Despite significant advancements in cancer therapies, mechanisms of resistance to treatments, particularly in advanced stages, remain incompletely understood [11,12]. Tumor cells frequently develop resistance to chemotherapy, limiting treatment efficacy and patient prognosis [13,14]. Although many studies have investigated this phenomenon, there is a pressing need for alternative strategies that sensitize tumor cells to treatment while sparing healthy tissues [14,15]. Identifying these mechanisms is crucial for enhancing therapeutic outcomes and overcoming the limitations of current oncological treatments [16,17,18,19].

Given the limitations of existing therapies, novel approaches such as caloric restriction (CR) hold promise. CR, defined as a sustained reduction in calorie intake without malnutrition, has been extensively studied for its benefits in extending lifespan, improving metabolic health, and reducing the risk of chronic diseases, including cancer [20,21]. However, its application as an adjunct cancer therapy remains underexplored, particularly in the context of advanced malignancies. Preclinical studies suggest that short-term CR, combined with chemotherapy, may enhance its efficacy while mitigating adverse effects, offering a potential strategy to overcome tumor resistance [22,23,24]. Despite this potential, questions remain regarding the long-term feasibility and mechanisms through which CR modulates tumor metabolism and immune response, particularly in aggressive cancers like sarcoma [25,26,27].

This study evaluated the safety, feasibility, and antitumoral effect of caloric restriction combined with doxorubicin in a preclinical Sarcoma-180 model. We specifically examined whether caloric restriction could enhance antitumor responses while mitigating the systemic toxicity typically associated with chemotherapy. The data obtained demonstrate that the combined regimen effectively reduced tumor volume and mass, accompanied by lower oxidative stress markers and increased antioxidant activity within the tumor.

At the systemic level, caloric restriction improved metabolic resilience. The intervention preserved body weight, reduced serum triglyceride levels, and attenuated hematological alterations commonly induced by doxorubicin. Immunological parameters were also improved, and markers of DNA damage in peripheral blood were reduced, indicating a decrease in off-target toxicity. Histological evaluation supported these results, revealing tumor features consistent with increased cell death and, in the liver, evidence of adaptive repair.

Together, these results indicate that caloric restriction enhances the antitumor effect of doxorubicin while offering protection to healthy tissues. This strategy demonstrates feasibility and safety, presenting a promising metabolic intervention to enhance chemotherapy efficacy and tolerability.

## 2. Materials and Methods

### 2.1. Animals and Ethical Principles

Twelve-week-old, 25 g Swiss mice were used (*n* = 5–10/group). They were housed in the Animal Facility II of the Federal University of Piauí, located in the Department of Biophysics and Physiology at the Center for Health Sciences. The facility provided an environment with controlled ventilation, humidity (40 ± 5%), and temperature (22 ± 2 °C). The room also had artificial lighting to maintain a 12 h light–dark cycle (lights on at 6 a.m.), and the animals had free access to filtered water and rat chow (NUVILAB CR-1, Quimtia, Colombo, PR, Brazil), except during the experimental period when food consumption was controlled.

Food intake and body weight gain were monitored throughout the entire experimental design. The animals were housed in individual plastic cages, lined with bedding, and covered with a metal grate. This study was submitted to and approved by the Ethics Committee on Animal Use (CEUA) of the Federal University of Piauí (UFPI), under protocol number 752/2022, following the principles adopted by the Brazilian College of Animal Experimentation (COBEA).

### 2.2. Sample Size and Statistical Power Calculation

The sample size required for the in vivo experiments was determined through a power calculation to ensure robust statistical interpretation of the results, following principles established for biomedical and behavioral studies. This calculation was essential to justify the number of animals per experimental group (AL, ALDOX, CR, CRDOX).

The minimum required sample size (*n*) per group was estimated using the following formula:

$$n\geq 1+[2C\times ({s/d)}^{2}]$$

where

- *n* represents the number of animals per group.
- *s* (maximum standard deviation or expected variation) was established at 0.2 (20%).
- *d* (minimum expected difference between groups, representing the effect size to be detected) was set at 0.5 (50%).
- *C* is the statistical factor defined by (zα + zβ)^2^.

The values for z were based on a 95% confidence interval, corresponding to a statistical significance level of *p* < 0.05. The calculation of the *C* factor was performed as follows: *C* = (1.96 + 1.282)^2^ = 10.51

Note: The value *zα* = 1.96 corresponds to a two-tailed significance level of 5% (*p* < 0.05), and *zβ* = 1.282 corresponds to a desired statistical power (1–*β*) of 80%, the generally accepted minimum threshold for biological studies.

Substituting the parameters into the sample size formula, the minimum number of animals required was calculated

$$n\geq 1+[2\times 10.51\times {\left(\frac{0.2}{0.5}\right)}^{2}]\;n\geq 4.3632$$

Rounding the result to the next whole number, the minimum required sample size for statistically significant results was *n* = 5 animals per group.

In the present study, we utilized 08 animals per group (AL, ALDOX, CR, and CRDOX). This size is consistent with established literature for experimental and behavioral testing, which often employs 8 to 10 animals per group. The final sample size of *n* = 8 is consistent with the power calculation (8 > 5), providing sufficient statistical power to detect the predefined biological effect (a 50% difference).

Due to the specific technical and volume requirements of certain assays, the final sample size (n per group) varied across the different measurements, as detailed in each figure legend. Specifically, the sample sizes used were *n* = 10 for Nutritional and Tumor parameters; *n* = 6–8 for Hematological, Biochemical, and Lipid Profile analyses; *n* = 6–8 for Oxidative Stress and Antioxidant Activity markers; *n* = 8 for Genotoxicity (Comet Assay), and Histopathological analysis; and *n* ranging from 5–7 for the Survival Analysis, with specific counts provided in the corresponding figure legend.

### 2.3. Tumor Implantation of Sarcoma-180 Cells and In Vivo Analysis

Initially, local trichotomy along the thoracoabdominal region of the mice was performed under anesthesia with xylazine hydrochloride (15 mg/kg i.m.) and ketamine hydrochloride (80 mg/kg i.p.). After dilution and counting of S-180 cells, a cell suspension of 4 × 10^6^ cells/mL in lactated Ringer’s solution plus 1% gentamicin was prepared. A 0.5 mL injection was administered into the left axillary region of the mice [28]. Body weights were determined daily, and tumor size was measured using a digital caliper. Tumor volume was calculated using the equation: tumor volume (mm^3^) = (length × width × height) × π/6, where length, width, and height are in millimeters.

### 2.4. Monitoring of Animals and Tumor Development

Body weights and food consumption were recorded daily, and tumor size was measured using a digital caliper. Tumor volume was calculated using the following equation: tumor volume (mm^3^) = (length × width × height) × π/6, where length, width, and height are in millimeters [29].

### 2.5. Determination of Basal Food Intake (AL Groups)

Prior to the experimental phase, the average daily food intake for the AL groups was meticulously determined over a period of seven consecutive days. This was accomplished by weighing the standard food pellets provided to the cages and reweighing the remaining pellets and any measurable spillage exactly 24 h later. The basal food intake of the AL animals served as the 100% control value for the calculation of Calorie Restriction (CR).

### 2.6. Caloric Restriction Protocol

The study involved four experimental groups: Ad Libitum (AL), Ad Libitum + Doxorubicin (ALDOX), Calorie Restriction (CR), and Caloric Restriction + Doxorubicin (CRDOX).

The caloric restriction protocol was implemented as a 40% reduction in the established average daily food intake for the AL control group. Specifically, animals in the CR and CRDOX groups received only 60% of the control group’s average daily intake. This restricted food allowance was provided once daily at a consistent time. This 40% reduction protocol was applied for a period of 10 days after sarcoma induction. Throughout the experimental period, the body weight of all individual mice and the residual food weight in the cages were monitored daily to ensure compliance with the 40% caloric restriction target and to detect any signs of unexpected distress, in accordance with ethical guidelines.

### 2.7. Experimental Protocol

The animals were randomly assigned to four experimental groups: Ad Libitum (AL), Ad Libitum + Doxorubicin (ALDOX), Caloric Restriction (CR), and Caloric Restriction + Doxorubicin (CRDOX). Doxorubicin was administered at 2 mg/kg of body weight i.p. [30,31].

**Figure 1:**
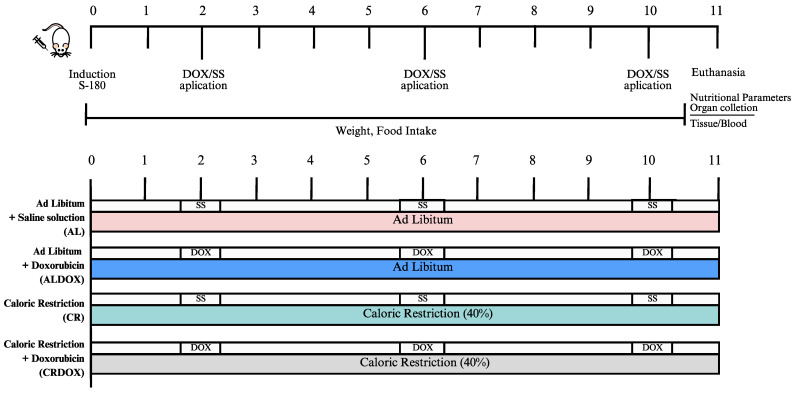
Experimental Design and Treatment Protocol in S180-Induced Tumor-Bearing Mice. S180 cells were inoculated on day 0; DOX or saline was administered on days 3, 6, and 10. Body weight and food intake were monitored daily. Euthanasia and sample collection occurred on day 11. Groups: AL: ad libitum + saline; ALDOX: ad libitum + doxorubicin; CR: caloric restriction (40%) + saline; CRDOX: caloric restriction (40%) + doxorubicin. Abbreviations: DOX: doxorubicin; SS: saline solution; CR: caloric restriction.

The animals were euthanized using an overdose of anesthetic (sodium thiopental—150 mg/kg + lidocaine hydrochloride 10 mg/kg, i.p.) or ketamine 10% (Dopalen) 100 mg/kg and xylazine 2% (Anasedan), followed by cervical dislocation. After the experimental procedures, the animals were placed in plastic bags and stored in a freezer (−20 °C) at the Department of Biophysics and Physiology of UFPI. The carcasses were later collected by a company contracted by the university, certified for the proper disposal of animal remains (Figure 1).

### 2.8. Nutritional Characterization

Nutritional assessment was carried out through food consumption and animal weight monitoring to analyze changes in body weight.

### 2.9. Biochemical and Hematological Analysis

Total blood collection for biochemical and hematological analyses was performed via intracardiac puncture. The collected blood was divided into separate tubes containing different anticoagulants: one with heparin and another with EDTA. The material collected in tubes containing heparin was centrifuged for plasma separation, and 120 µL of plasma was set aside for analysis of gamma-glutamyl transferase (GGT), glutamic-pyruvic transaminase (GPT), and glutamic-oxaloacetic transaminase (GOT) activity using Labtest kits (Labtest Diagnóstica, Lagoa Santa, MG, Brazil), as specified by catalog numbers 105, 108, and 109, respectively. Additionally, tests were conducted using Labtest kits to determine triglycerides, total cholesterol, high-density lipoprotein (HDL), and low-density lipoprotein (LDL), according to catalog numbers 87, 76, 145, and 146, respectively, and absorbances were measured on a Polaris spectrophotometer with wavelengths ranging from 340 to 700 nm, as specified in each kit’s manual. For hematological analysis, 1 mL of whole blood was applied to the SYSMEX^®^ hematological analyzer (model XS-1000i, Sysmex Corporation, Kobe, Japan), which uses fluorescent flow cytometry as its method. The investigated parameters included hematocrit, hemoglobin, mean corpuscular hemoglobin concentration (MCHC), red cell distribution width (RDW), total erythrocytes, total leukocytes, and platelet count.

### 2.10. Evaluation of Oxidative Stress Markers and Antioxidant Activity

#### 2.10.1. Evaluation of Nitrite/Nitrate (NOx)

Nitric oxide production in tumor tissue was indirectly assessed by measuring nitrate (NO_3_^−^) and nitrite (NO_2_^−^) levels (NOx) using the Griess assay. Samples were homogenized in 0.15 M KCl, centrifuged, and 100 μL of the supernatant was mixed with 100 μL of Griess reagent (phosphoric acid, sulfanilamide, and N-(1-naphthyl)ethylenediamine dihydrochloride). After 10 min, the absorbance was read at 540 nm, and the results were expressed as μmol NOx [32,33].

#### 2.10.2. Malondialdehyde (MDA) Concentration

MDA levels were determined according to Uchiyama and Mihara [34] and Da Silva et al. [35], using 1,1,3,3-tetraethoxypropane (TEP) as a standard. Tumor tissue was homogenized in 0.15 M KCl, centrifuged, and 200 μL of the supernatant was reacted with acetic acid and 0.5% thiobarbituric acid (TBA, pH 3.5). After incubation at 85 °C for 1 h and cooling, absorbance was measured at 532 nm. Results were expressed as nmol MDA/mL.

#### 2.10.3. Evaluation of Myeloperoxidase (MPO) Activity

MPO activity was determined as described by Severo et al. [36]. Tumor tissues were homogenized in 0.5% hexadecyltrimethylammonium bromide (HTAB) buffer, centrifuged, and the resuspended pellet was incubated with o-dianisidine and H_2_O_2_. Absorbance was read at 450 nm, and results were expressed as U MPO/mg tissue.

#### 2.10.4. Evaluation of Reduced Glutathione (GSH) Activity

GSH levels were determined by measuring non-protein sulfhydryl groups (NPSH) [36]. Tumor tissue homogenates in 0.02 M EDTA were deproteinized with trichloroacetic acid, centrifuged, and the supernatant was reacted with Tris buffer and DTNB. Absorbance was read at 412 nm, and results were expressed as μg GSH/g tissue.

#### 2.10.5. Evaluation of Superoxide Dismutase (SOD) Activity

SOD activity was measured according to the method described by Severo et al. [36]. Tumor tissue homogenates were incubated with phosphate buffer containing L-methionine, Triton X-100, hydroxylamine chloride, and EDTA, followed by riboflavin. After incubation, Griess reagent was added, and absorbance was read at 550 nm. Results were expressed as U SOD/μg tissue.

### 2.11. Alkaline Comet Assay

The comet assay was performed using peripheral blood to analyze the genotoxic profile [37,38]. Aliquots of 10 μL of peripheral blood were mixed with a thin layer of low-melting-point agarose (0.75%) (90 μL) and placed on slides pre-coated with 1.5% ultrapure agarose. The slides were immersed in a lysis solution (2.5 M NaCl, 100 mM EDTA, 10 mM Tris, pH 10) containing 1% Triton X-100 and 10% DMSO, which was added at the time of use, for up to 72 hours at 4 °C. The slides were then incubated in an alkaline buffer (300 mM NaOH, 1 mM EDTA, pH > 13) for 20 min and exposed to an electric current of 300 mA and 25 V (0.90 V/cm) for an additional 20 min in an electrophoresis apparatus. After electrophoresis, the slides were neutralized with 0.4 M Tris (pH 7.5) and stained with silver solution. The damage index (DI) was calculated using the formula DI = Σ (number of cells in a given damage class × damage class), ranging from 0 to 400.

### 2.12. Histological Analysis

#### Hematoxylin and Eosin Staining

After euthanasia, tumors were removed and stored in 10% formalin for 48 h before processing for histopathological studies. Tumors and organs were placed in cassettes and processed automatically using a Leica TP1020 Automatic Tissue Processor (Leica Biosystems, Nussloch, Germany). The samples were embedded in paraffin, sectioned using a microtome (Leica Biosystems, Nussloch, Germany) at a thickness of 5 µm, and stained with hematoxylin and eosin (H&E). The slides were coded and examined by a pathologist blinded to the groups. Histopathological alterations were assessed in sections from at least eight mice (*n* = 8) per group [39,40].

### 2.13. Survival Test

The survival time of the mice was recorded from the eleventh day of the experiment until death. The survival rate was calculated and compared between the groups.

### 2.14. Statistical Analysis

The data are presented as means ± standard error of the mean (SEM) and analyzed using GraphPad Prism software, version 8.0 (GraphPad Prism Software, Boston, MA, USA). The Kolmogorov–Smirnov test was used to assess the normality of the data. One-way or two-way ANOVA followed by Tukey’s test was applied to compare variables with a normal distribution, while the Kruskal–Wallis test was used for non-parametric data. The survival rate between groups was described using Kaplan–Meier curves. Differences were considered significant when *p* < 0.05, with a 95% confidence interval. To ensure statistical rigor and transparency, 95% confidence intervals (CI) are explicitly reported for key findings in the results section, along with the means ± SEM. Furthermore, effect sizes are quantified and reported throughout the Results section using percentage differences to describe the magnitude of the effect of the interventions.

## 3. Results

### 3.1. Caloric Restriction Combined with Chemotherapy Demonstrated Superior Antitumor Activity Compared with Doxorubicin Alone, Without Compromising Body Weight

**Figure 2:**
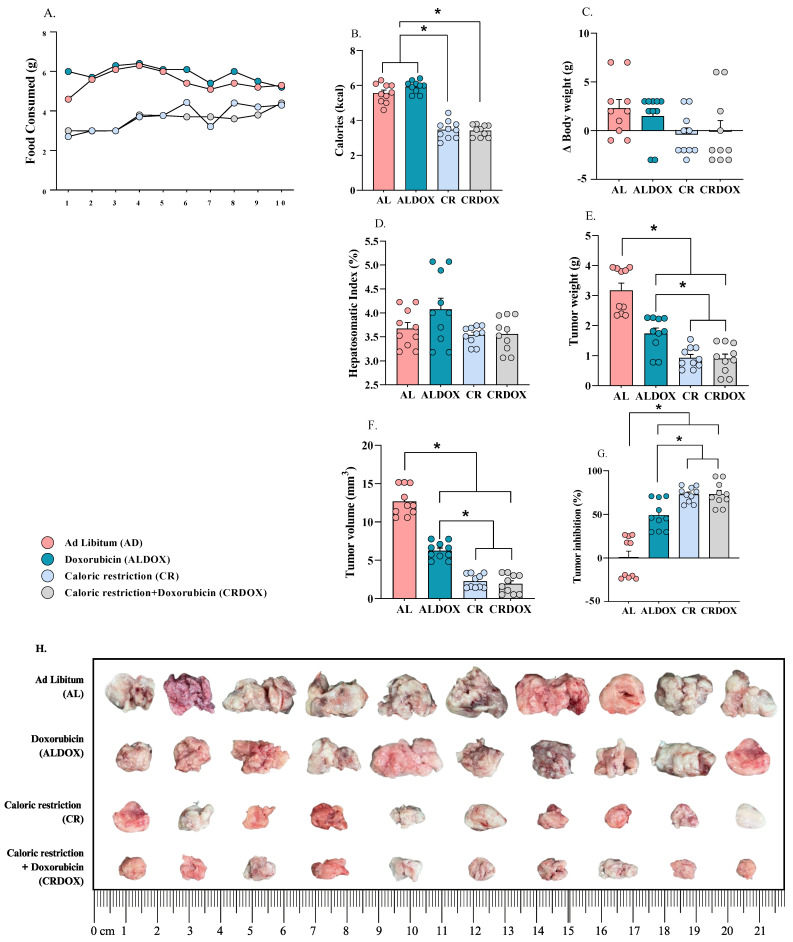
Caloric restriction combined with doxorubicin more effectively suppresses tumor growth without compromising body weight. (**A**,**B**) Reduction in food intake and caloric consumption between AL/ALDOX and CR/CRDOX groups. (**C**) Δ body weight change (g). (**D**) Hepatosomatic index (HSI = (liver weight/body weight) × 100). (**E**) Tumor mass. (**F**) Tumor volume. (**G**) Tumor inhibition. (**H**) Representative images demonstrating the size difference in S-180 tumor tissues. Data expressed as mean ± SEM and analyzed by one-way ANOVA test, followed by Tukey’s test. Significance: * *p* < 0.05, *n* = 10 per group. Groups: AL: ad libitum + saline; ALDOX: ad libitum + doxorubicin; CR: caloric restriction (40%) + saline; CRDOX: caloric restriction (40%) + doxorubicin. Abbreviations: DOX: doxorubicin; SS: saline solution; CR: caloric restriction.

Caloric restriction combined with doxorubicin (CRDOX) (Figure 2A,B) promoted antitumor activity, as evidenced by reductions in tumor mass (Figure 2E,H) and volume (Figure 2F), as well as an increase in the percentage of tumor inhibition (Figure 2G). The groups subjected to caloric restriction (CR and CRDOX) showed no differences in body weight after treatment when compared with animals fed ad libitum (AL and ALDOX) (Figure 2C,D). This finding suggests that caloric restriction is safe and well-tolerated, particularly in terms of preserving body composition. Considering that maintaining body composition is critical in oncologic nutrition, as excessive weight loss can negatively impact patient prognosis, these results further support the antitumor potential of caloric restriction in the sarcoma-180 model, highlighting its potential application as a therapeutic strategy in cancer treatment. 

### 3.2. Caloric Restriction Protects Against Doxorubicin-Induced Hematological and Immune Toxicity in the Sarcoma-180 Model

**Figure 3:**
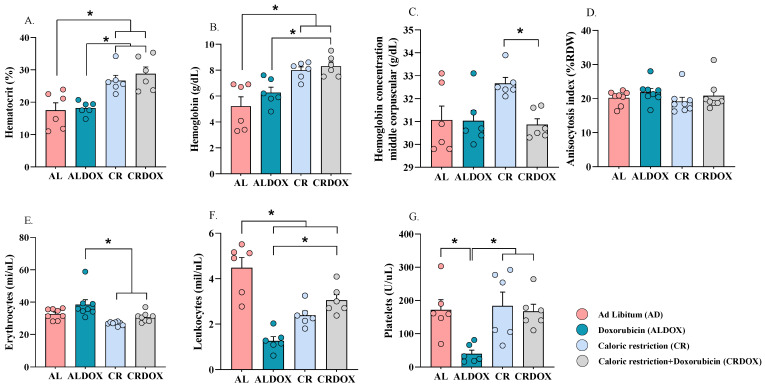
Combined caloric restriction and doxorubicin enhance hematological and immune profiles in the sarcoma-180 model. (**A**) Hematocrit: Percentage of volume occupied by red blood cells or erythrocytes in the total blood volume (*n* = 6 per group). (**B**) Hemoglobin concentrations (*n* = 6 per group). (**C**) Mean Corpuscular Hemoglobin Concentration (MCHC) (*n* = 6 per group). (**D**) Red cell distribution width (RDW) (*n* = 8 per group). (**E**) Erythrocytes (*n* = 8 per group). (**F**) Leukocytes (*n* = 6 per group). (**G**) Platelets (*n* = 6 per group). Data expressed as mean ± SEM and analyzed by one-way ANOVA, followed by Tukey’s test. Significance: * *p* < 0.05. Groups: AL: ad libitum + saline; ALDOX: ad libitum + doxorubicin; CR: caloric restriction (40%) + saline; CRDOX: caloric restriction (40%) + doxorubicin. Abbreviations: DOX: doxorubicin; SS: saline solution; CR: caloric restriction.

Caloric restriction (CR) provided protection against chemotherapy-induced systemic damage, as evidenced by the preservation of key hematological parameters. Specifically, the combined CRDOX treatment resulted in a substantial 58.05% increase in hematocrit (Figure 3A) and a 32.58% increase in hemoglobin levels (Figure 3B) compared to chemotherapy administered alone (ALDOX). These findings strongly suggest that CR exerts a protective effect against chemotherapy-induced hematotoxicity, thereby contributing to the preservation of blood cells. Interestingly, a decrease in erythrocyte counts was observed in the CR and CRDOX groups compared to the ALDOX group (Figure 3E), while the anisocytosis index (Figure 3D) remained stable across all groups. The mean corpuscular hemoglobin concentration (MCHC) (Figure 3C) in the CR group differed from the CRDOX group, showing an increase in the group subjected only to the dietary intervention. Finally, the combined treatment also increased leukocyte (Figure 3F) and platelet (Figure 3G) counts compared with chemotherapy alone.

### 3.3. Caloric Restriction Synergizes with Doxorubicin to Induce Hepatic Adaptations and Reduce Triglyceride Levels in the Sarcoma-180 Model

**Figure 4:**
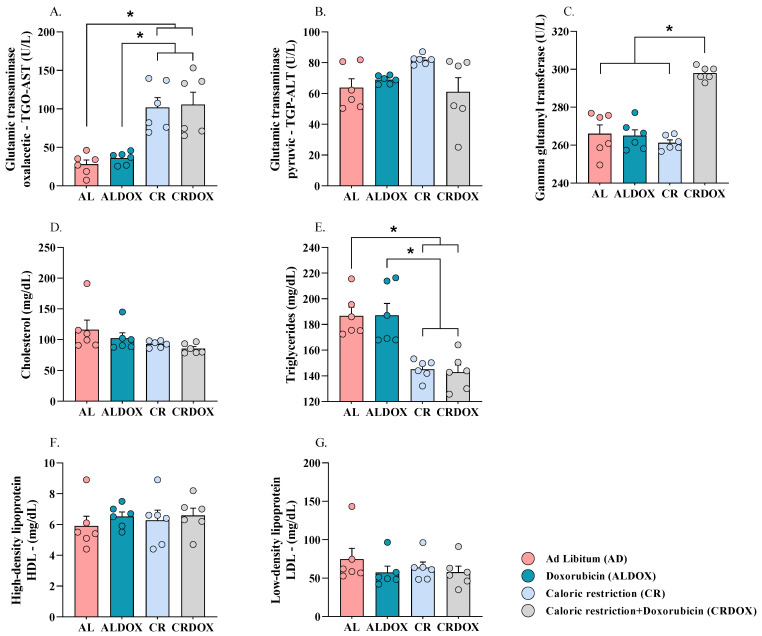
Association between caloric restriction and doxorubicin promotes hepatic adaptations and reduces triglyceride levels in Sarcoma-180–bearing mice. (**A**) Concentration of TGO-AST (*n* = 6 per group). (**B**) TGP-ALT (*n* = 6 per group). (**C**) Gamma-glutamyl transferase (*n* = 6 per group), and lipid profile: (**D**) Cholesterol (*n* = 6 per group). (**E**) Triglycerides (*n* = 6 per group). (**F**) HDL (*n* = 8 per group). (**G**) LDL (*n* = 6 per group). Data expressed as mean ± SEM and analyzed by one-way ANOVA followed by Tukey’s test. Significance: * *p* < 0.05. Groups: AL: ad libitum + saline; ALDOX: ad libitum + doxorubicin; CR: caloric restriction (40%) + saline; CRDOX: caloric restriction (40%) + doxorubicin. Abbreviations: DOX: doxorubicin; SS: saline solution; CR: caloric restriction.

It was observed that caloric restriction, either alone or in combination with chemotherapy, promoted specific alterations in hepatic biochemical markers and significantly reduced circulating lipid concentrations. Specifically, an increase in glutamic transaminase oxalacetic (TGO-AST) levels (Figure 4A) was detected in the CR and CRDOX groups compared with the AL and ALDOX groups. In contrast, glutamic transaminase pyruvic (TGP-ALT) levels (Figure 4B) and total cholesterol levels (Figure 4D) remained unchanged across all treatments. Regarding gamma-glutamyl transferase (GGT) (Figure 4C), the CRDOX group exhibited a significant elevation relative to the other groups, indicating greater hepatic enzymatic activity in animals subjected to nutrient restriction.

However, a significant reduction in triglyceride concentrations (Figure 4E) was observed in the CR and CRDOX groups compared with the AL and ALDOX groups. For high-density lipoprotein (HDL) and low-density lipoprotein (LDL) (Figure 4F,G), no statistically significant differences were identified. It is important to note that changes in hepatic biochemical markers, such as TGO-AST and GGT, may occur even in the presence of histological findings consistent with tissue repair.

### 3.4. Caloric Restriction, Alone or Combined with Doxorubicin, Reduces Oxidative Stress Markers and Increases Superoxide Dismutase

**Figure 5:**
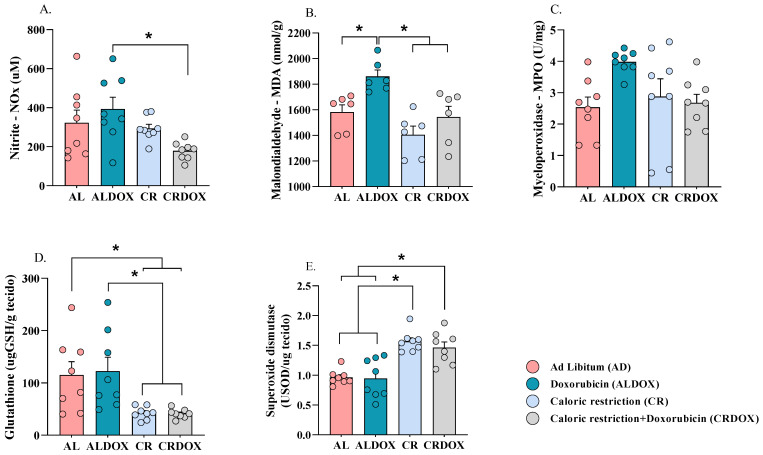
Caloric restriction combined with doxorubicin reduces oxidative damage by enhancing antioxidant responses. (**A**) Nitrites (NOx) (*n* = 8 per group), (**B**) Malondialdehyde (MDA) (*n* = 6 per group), (**C**) Myeloperoxidase (MPO) (*n* = 8 per group), (**D**) Glutathione (GSH) (*n* = 8 per group), (**E**) Superoxide dismutase (SOD) (*n* = 8 per group). Data are expressed as mean ± SEM and analyzed by one-way ANOVA, followed by Tukey’s test. Significance: * *p* < 0.05. Groups: AL: ad libitum + saline; ALDOX: ad libitum + doxorubicin; CR: caloric restriction (40%) + saline; CRDOX: caloric restriction (40%) + doxorubicin. Abbreviations: DOX: doxorubicin; SS: saline solution; CR: caloric restriction.

The results demonstrate that caloric restriction (CR), alone or combined with doxorubicin (CRDOX), reduces oxidative stress in the tumor and increases antioxidant activity. Specifically, the combined treatment reduced nitrite (NOx) levels by 54.54% (Figure 5A) and malondialdehyde (MDA) levels by 17% (Figure 5B) compared to chemotherapy alone. Myeloperoxidase (MPO) activity (Figure 5C) remained unchanged between groups. Furthermore, both the combined treatment and CR alone increased superoxide dismutase (SOD) activity by 54.54% (Figure 5E). However, a 66.45% reduction in reduced glutathione (GSH) levels (Figure 5D) was also observed in the combined treatment.

### 3.5. Caloric Restriction Combined with Chemotherapy Provides a Protective Effect Against Chemotherapy-Induced Genotoxic Damage

**Figure 6:**
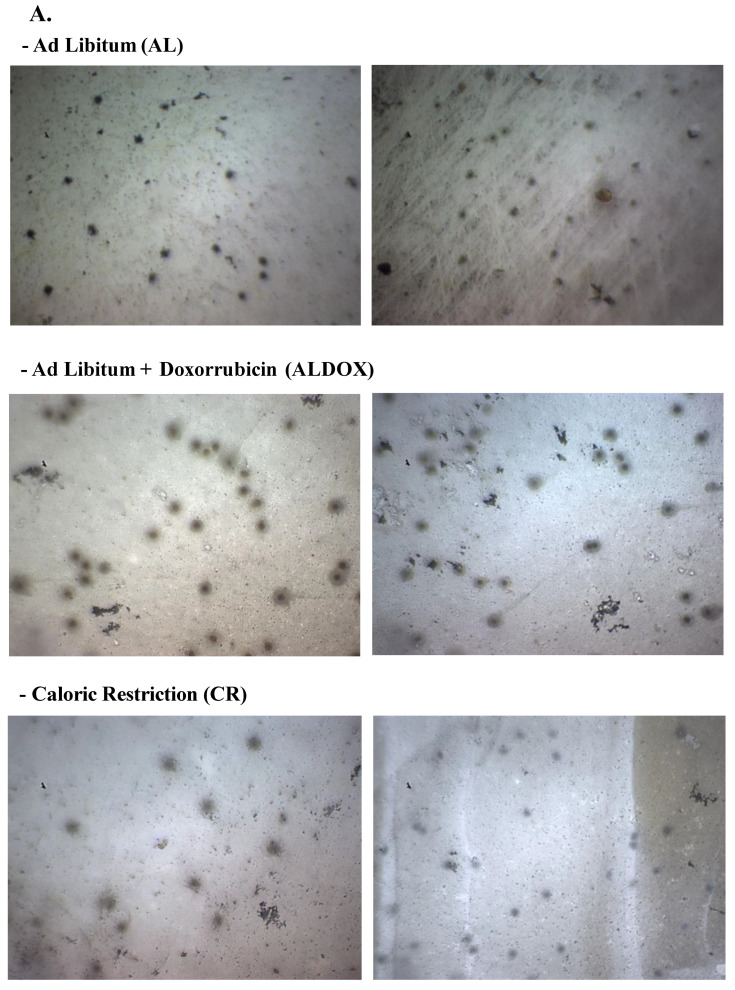

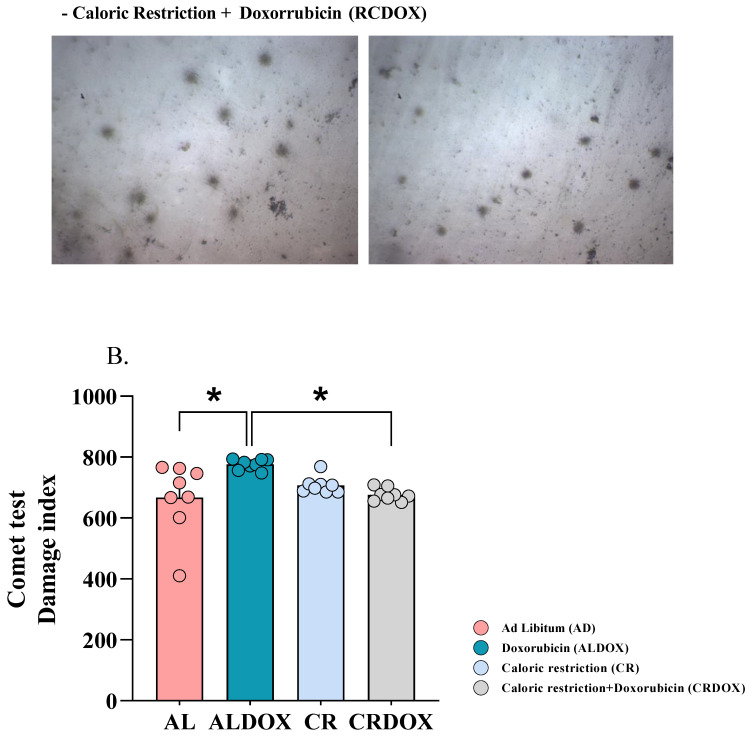
Caloric restriction combined with doxorubicin reduces DNA damage compared with doxorubicin alone. (**A**) Representative micrographs of the Comet Assay (captured at 10x magnification), where DNA damage was measured by the amount and distance of DNA migration away from the nucleus, indicative of DNA strand breaks. Higher values correspond to greater damage. (**B**) Biomarker assay for DNA damage in blood cells. Data are expressed as mean ± SEM and analyzed by one-way ANOVA, followed by Tukey’s post hoc test. Significance: * *p* < 0.05, *n* = 8 per group. Groups: AL: ad libitum + saline; ALDOX: ad libitum + doxorubicin; CR: caloric restriction (40%) + saline; CRDOX: caloric restriction (40%) + doxorubicin. Abbreviations: DOX: doxorubicin; SS: saline solution; CR: caloric restriction.

Caloric restriction combined with chemotherapy (CRDOX) reduced DNA damage in peripheral blood cells. These findings support the hypothesis that integrating caloric restriction with chemotherapy decreases cellular susceptibility to treatment-induced genotoxicity (Figure 6).

### 3.6. Histological Analysis of Tumor and Hepatic Tissue from the CRDOX Combination Group Demonstrates Control of Cellular Invasion, Apoptosis, and Immune Response

**Figure 7:**
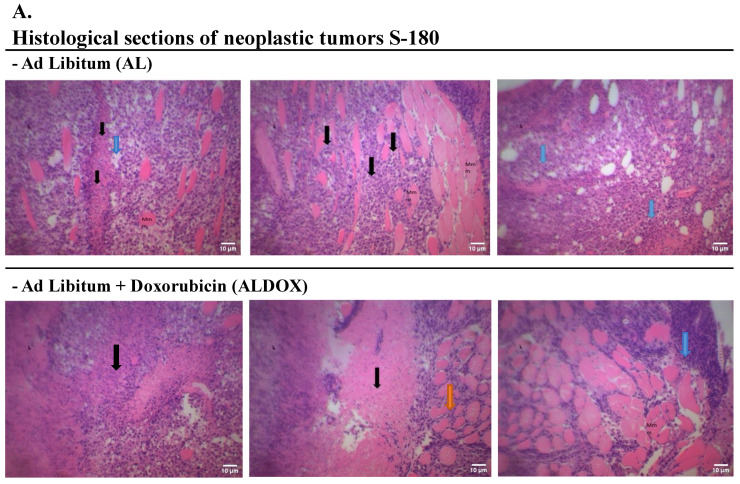

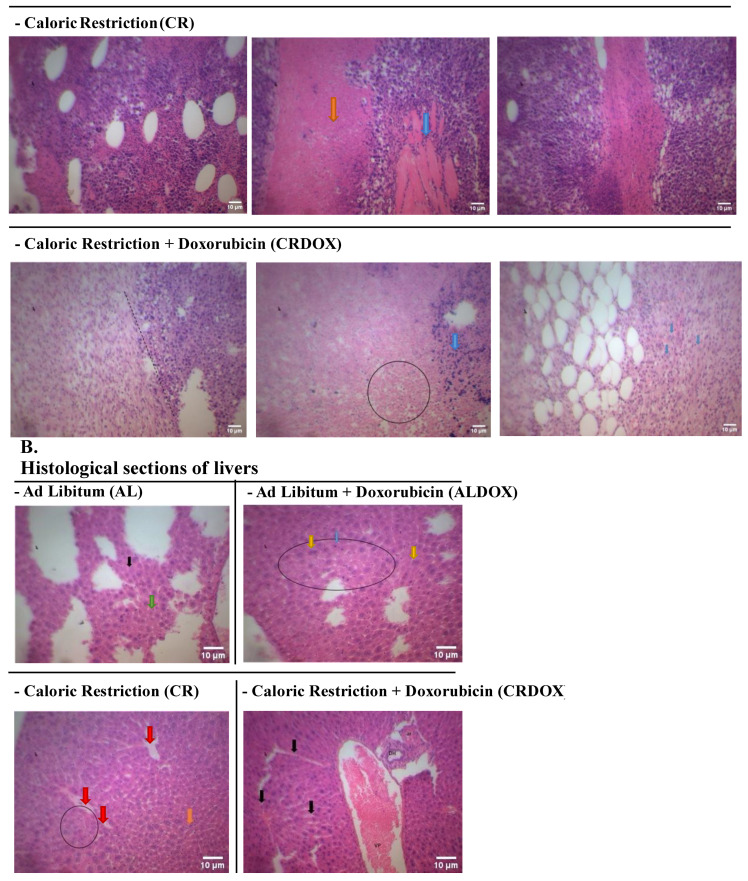
Caloric restriction combined with doxorubicin induces histological changes compatible with antitumor activity in Sarcoma-180 tumors and promotes adaptive repair features in the liver. (**A**) Histological sections of sarcoma-180 tumors from mice, stained with eosin and hematoxylin. Black arrow: invasion. Blue arrow: necrosis, apoptosis, and pyknotic nuclei. Orange arrow: eosinophilia. Circle: anucleate eosinophilic area. (**B**) Histological sections of mice livers bearing Sarcoma-180, stained with eosin and hematoxylin. Black arrow: anucleated hepatocytes. Yellow arrow: binucleated hepatocytes. Green arrow: lymphocytes. Red arrow: dilated sinusoids. Circle: pyknotic nuclei (H&E, magnification of 40×). Scale bar = 10 µm. Groups: AL: ad libitum + saline; ALDOX: ad libitum + doxorubicin; CR: caloric restriction (40%) + saline; CRDOX: caloric restriction (40%) + doxorubicin. Abbreviations: DOX: doxorubicin; SS: saline solution; CR: caloric restriction.

Histological sections of tumors with distinct characteristics were analyzed, presenting high cellular density and a peculiar architecture. Black arrows highlight the pronounced invasion of these cells into the adjacent muscular tissue, resulting in a significant loss of normal architecture. In areas indicated by blue arrows, phenomena of necrosis, where the tissue underwent degeneration, and apoptosis were observed, adding complexity to the pathological picture. Additionally, regions of eosinophilia, indicated by orange arrows, demonstrate increased migration of eosinophils to the site of the inflammatory process or the prolonged survival of these cells in the tissue (Figure 7).

In the CRDOX group, we observed eosinophilic tissue with undefined architecture, more fibrous and less cellular on the left, while on the right, we observed a more densely cellular tissue with well-stained nuclei, representing a transition between an area of intense tumor activity and tumor reduction. We also identified cellular remnants with pyknotic nuclei, characterized by extremely condensed chromatin due to the pathological process, indicated by the blue arrows. Within the circle, a large anucleate eosinophilic area was identified, characterized by the death of tumor cells and empty spaces that indicate localized tissue loss (Figure 7).

The massive destruction of hepatocytes resulted in the loss of liver lobular architecture. The empty spaces observed indicate the loss of hepatocytes during the histological process, possibly due to the fragility of cell junctions. Additionally, the presence of lymphocytes, indicated by the green arrow, suggests an immune response at the site. Several anucleated hepatocytes, indicated by the black arrow, and areas of hepatic degeneration, evidenced by nuclear loss and pyknotic nuclei, indicated in the circle, were observed. We also identified binucleated hepatocytes, indicated by the yellow arrows. In the CRDOX group, we also observed the portal space, highlighting the portal vein branch, arteriole, and hepatic duct.

### 3.7. Caloric Restriction Combined with Chemotherapy Revealed Increased Survival in Sarcoma-Bearing Animals

**Figure 8:**
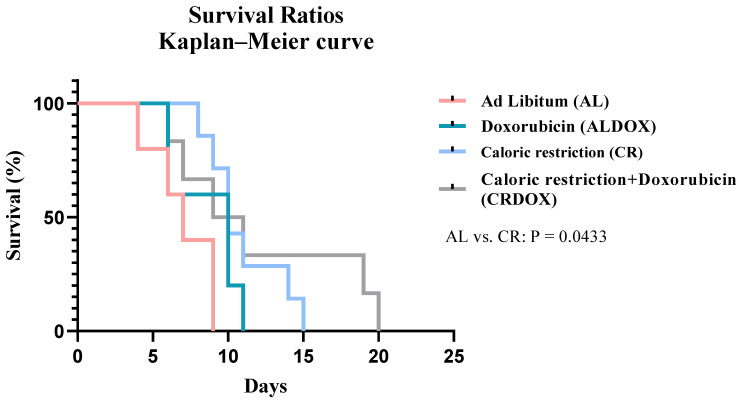
Survival proportions and Kaplan–Meier curve compare the survival of the AL (*n* = 5), ALDOX (*n* = 5), CR (*n* = 7), and CRDOX (*n* = 6) groups, where the Log-Rank test indicated a statistical difference between the groups. Significance: ALDOX: ad libitum + doxorubicin; CR: caloric restriction (40%) + saline; CRDOX: caloric restriction (40%) + doxorubicin. Abbreviations: DOX: doxorubicin; SS: saline solution; CR: caloric restriction.

The combined therapy demonstrated significant efficacy in prolonging the survival of tumor-bearing animals, emerging as a promising approach compared to isolated treatments. The median survival was 10 days for the combined treatments (ALDOX, CRDOX) and Caloric restriction (CR), in contrast to 7 days observed for the standard group (AL). Caloric restriction improved survival by approximately 81.82% compared to standard treatment (Figure 8).

## 4. Discussion

Nutritional management in oncology often raises concerns about weight loss during treatment since malnutrition is associated with increased mortality and disease progression [41,42,43,44,45]. However, our results show that 40% CR can be safely applied, expanding the therapeutic window of this intervention in the experimental sarcoma 180 model, as it preserved the animal’s body weight.

The evidence suggests that CR, either alone or combined with chemotherapy, significantly reduced tumor weight and volume and increased the tumor inhibition rate while protecting normal cells from chemotherapy-induced damage, promoting protection against hematological and immune cells and DOX-associated genotoxicity. These findings highlight the phenomenon of differential stress resistance (DSR), characterized by the ability of caloric restriction to sensitize cancer cells to chemotherapy while protecting healthy cells [46].

Importantly, the strong antitumor activity of caloric restriction alone was evident in our model. CR frequently performed comparably to the combined treatment and surpassed the chemotherapy-alone group, achieving more than 50% tumor inhibition. This high efficacy is notable, as CR alone increased animal survival by approximately 81.82% compared to the ad libitum group (AL), reaching the same median survival time of 10 days observed in the combined CRDOX treatment. The strict 40% reduction in caloric intake induced a profound metabolic stress in the tumor microenvironment, compromising essential anabolic and proliferative processes required for tumor progression. Moreover, CR alone significantly reduced serum triglyceride levels and increased superoxide dismutase (SOD) activity within the tumor, indicating an intrinsic capacity to modulate oxidative stress and enhance antioxidant defenses. Histological analyses further supported these findings, revealing immune-mediated suppression in the tumor microenvironment driven by CR-induced metabolic remodeling.

CR promotes metabolic reprogramming by inducing a state of low energy availability that sensitizes tumor cells, which, according to the literature, is due to the inhibition of crucial oncogenic pathways such as IGF-1 and insulin signaling [21,47,48]. Previous studies suggest that this modulation results in the inhibition of the AKT–mTOR axis, contributing to the reversal of chemotherapy resistance [49,50,51,52,53]. In our study, CR and its combination with chemotherapy resulted in more than 50% tumor inhibition, demonstrating its ability to increase treatment responsiveness and overcome resistance mechanisms, representing a significant advancement in the development of metabolic therapies [54,55,56].

Chemotherapy often causes hematological and immunological toxicity due to its low selectivity [57,58]. The present results indicate that CR increases chemotherapy selectivity by modulating immune responses and reducing immunosuppressive factors while preserving hematological parameters. This modulation of the immune system is associated with better prognosis and greater therapeutic efficacy, reinforcing the potential of CR to synergize with anticancer approaches [58,59].

Additionally, CR plays an important role in DNA repair mechanisms. Genotoxic damage induced by chemotherapy can lead to cell cycle arrest and apoptosis [60,61,62,63]. Our study shows that CR combined with chemotherapy preserves blood cell counts while maintaining tumor suppression, suggesting that CR contributes to DNA damage repair in non-tumor tissues [64,65].

CR also reprograms lipid metabolism, reducing serum levels of cholesterol and triglycerides. Malignant cells depend on fatty acid synthesis and renewal for energy production, proliferation, and signaling [27,53,66]. Thus, lipid limitation through CR-induced pathways compromises essential processes for tumor survival, contributing to the observed inhibition.

Histopathological analysis reinforces these findings. The association between CR and chemotherapy delayed tumor development and induced apoptosis, evidenced by pyknotic nuclei and extensive areas of necrosis. The presence of lymphocytes and eosinophils in the tumor microenvironment supports the role of CR in enhancing immune system-mediated suppression [67,68,69].

Furthermore, CR increased the animals’ survival, which is attributed to its ability to attenuate risk factors such as regulating lipid metabolism and preventing the development of chronic diseases, including type II diabetes, cardiovascular diseases, neurodegenerative disorders, and cancer [70]. Thus, our work highlights the role of CR in acting synergistically with chemotherapy, promoting antitumor effects and protection of healthy tissues, which translates into increased animal survival. However, we emphasize that, although these preliminary data are promising and consistent, our research group considers it essential to deepen the understanding of the molecular mechanisms related to these results. We explicitly acknowledge that this study relies on systemic phenotypic and biochemical data and does not include direct measurements of proposed molecular signaling pathways, such as IGF-1 levels or Western blotting analysis for *p*-AKT and p-mTOR, as well as autophagy markers. The absence of this direct molecular data represents a key limitation in the comprehensive validation of the underlying mechanisms, such as AKT–mTOR axis inhibition and Differential Stress Resistance (DSR).

## 5. Conclusions

This study demonstrates that caloric restriction (CR), combined with doxorubicin (CRDOX), is a safe and feasible strategy in the Sarcoma-180 model, preserving body weight and avoiding metabolic disturbances. The combined regimen achieved a marked reduction in tumor volume and mass and promoted histological changes compatible with apoptosis and cell death.

These findings strongly support the concept of Caloric Restriction-Associated Differential Stress Resistance (CR-DSR). Systemically, CRDOX mitigated hematotoxicity and reduced chemotherapy-induced DNA damage in peripheral blood cells, confirming a protective effect on normal tissues. Furthermore, the intervention enhanced local antioxidant activity (SOD) while reducing oxidative stress markers (NOx and MDA) within the tumor.

In conclusion, CR enhanced the antitumor response, improved treatment tolerance, and resulted in significantly prolonged survival, positioning it as a promising metabolic strategy to increase the efficacy and safety profile of chemotherapy.

## Data Availability

The data supporting the findings of this study are available from the corresponding author upon reasonable request. Due to ethical restrictions related to the use of laboratory animals and institutional regulations, the datasets generated and analyzed during the current study are not publicly available. No publicly archived datasets were used in this manuscript.

## Abbreviations

The following abbreviations are used in this manuscript:

AL	Ad Libitum
ALDOX	Ad Libitum plus Doxorubicin
CR	Caloric Restriction
CRDOX	Caloric Restriction plus Doxorubicin
S180	Sarcoma-180
DOX	Doxorubicin
NOx	Nitrite/Nitrate
MDA	Malondialdehyde
MPO	Myeloperoxidase
GSH	Reduced Glutathione
SOD	Superoxide Dismutase
AST	Aspartate Aminotransferase
ALT	Alanine Aminotransferase
GGT	Gamma-Glutamyl Transferase
HDL	High-Density Lipoprotein
LDL	Low-Density Lipoprotein
EDTA	Ethylenediaminetetraacetic Acid
HTAB	Hexadecyltrimethylammonium Bromide
DTNB	5,5′-Dithiobis(2-nitrobenzoic acid)
SEM	Standard Error of the Mean
H&E	Hematoxylin and Eosin
DNA	Deoxyribonucleic Acid
DSR	Differential Stress Resistance
